# Taxation strategies for the governance of digital business model—An example of China

**DOI:** 10.3389/fpsyg.2022.1013228

**Published:** 2022-09-22

**Authors:** Yi Guo, Tingting Zou, Ziwei Shan

**Affiliations:** ^1^School of International Economics and Management, Beijing Technology and Business University, Beijing, China; ^2^School of Economics, Beijing Technology and Business University, Beijing, China.

**Keywords:** tax strategy management, digital business model, operations, digital ecosystem, digital technology

## Abstract

The digital business model emerges as a new business model and gradually penetrates global industries, and countries are putting in place various digital strategies to support their development. As one of the important tools, taxation strategies are highly expected by countries, which not only describe the economic development pattern of a country but also show the digital leadership of a country. Some countries have introduced their own unilateral digital services tax to govern their digital business models, while others have looked more to the global minimum tax, resulting in the current situation of both a unilateral digital services tax and a global minimum tax. However, both of them are of great reference value for the tax governance of digital business models. This paper compares the development history of digital tax strategies, categorizes, and analyzes the design logic of existing digital tax strategies, and takes China, one of the major digital economy countries, as an example to propose China’s digital tax strategies by drawing on international experience. We set an example for the design of digital economy tax strategies for countries around the world so that they can manage digital business models more efficiently.

## Introduction

The rapid development of information technology such as the Internet has spawned a series of new business models that take data resources as key production factors, modern information networks as an important carrier, and the effective use of information and communication technology as an efficiency improvement, and an important driving force for economic structure optimization. These new business models are gradually expanding and penetrating into all areas of the national economy (National Bureau of Statistics, 2018), benefiting economic growth by their speed, increasing marginal benefits, high penetration, sustainability, and external economy compared to traditional economic activities (Polevoi and Mamai, 2022; Wu and Yang, 2022), but their “no physical permanent establishment,” high concealment of economic activities, huge profitability and monopoly tendency. The characteristics of “no physical permanent establishment,” highly secretive economic activity, huge profitability, and monopolistic tendencies also bring a lot of uncertainties for stable economic growth.

In order to better exploit the role of the digital economy in the country’s economic growth, countries have introduced a variety of policies in an attempt to effectively control it. Tax policies are expected to be one of the most important tools for governments to manage their economies. The traditional taxation principle states that “profits are taxed where economic activity occurs and where value is created.” Under this principle, the intangibility of factors of production, the virtual nature of permanent establishments, and the digital business model makes it less likely that the “place of economic activity” and the “place of value creation” are the same, making it more difficult to tax their profits (Cui, 2019). At this point, there is a relatively unanimous consensus in the international community on the introduction of a digital services tax to address the challenges posed by the digital economy to the traditional international tax system.

However, the different levels of development of the digital economy make different countries have different ideas on their digital service tax bases, starting points, and tax rates. Nevertheless, as far as the digital economy itself is concerned, its unique cross-territory characteristics objectively require a relatively unified global tax governance system, which requires a consensus among different countries, otherwise, it is likely to give rise to tax pits, tax havens, and other phenomena that intensify bottom-up competition in international taxation and undermine the fairness of the international business environment (Cassee, 2019). This has given rise to the coexistence of two tax governance strategies for the digital economy in the international community. One is the unilateral digital services tax, which reflects the differentiated digital economy development characteristics of each country, and is a tax imposed by each country on the digital products and services provided by digital enterprises, including direct unilateral digital services tax and indirect digital services tax in the form of the original tax. The other is a global minimum tax on the characteristics of the digital economy itself, which is introduced as an important element of Pillar 2 of the two-pillar proposal for a two-pillar framework on base erosion and profit loss (BEPS), requiring member countries to levy at least a uniform effective rate of corporate income tax on eligible multinational companies in their territories (OECD, 2021a,b,c).

Several countries have publicly stated that they will repeal their unilateral digital service tax proposals after the global minimum tax is introduced. As of November 2021, over 135 countries and jurisdictions, including China, have joined the global minimum tax proposal (OECD, 2021a,b,c). The BEPS Inclusive Framework announced a 15% global minimum tax to be implemented by the end of 2023 (OECD, 2021a,b,c), but the exact timing of implementation and profit-sharing principles were not clarified and member states were unable to agree on the obligations to be borne by the introduction of a global minimum tax, particularly in countries with fewer parent companies such as Estonia, Poland, and Sweden.

China has the second-highest level of digital economy development in the world, and the country is introducing various policies to improve digital infrastructure construction, support digital technology innovation and the development of digital enterprises, and strive to be a provider of digital products and services (China Academy of Information and Communication Research, 2021). The introduction of the global minimum tax is closely related to this development goal, so China should actively participate in the formulation of the global minimum tax as a digital leader to exclude the threats that are unfavorable to the development goals of China’s digital economy while maintaining the trend of economic globalization. The real economy is the lifeblood and core of our national economy, and the linking effect and digital empowerment of the digital economy can greatly improve the efficiency of the industrial chain, which may become a turning point for a new stage of our real economy (Li et al., 2022; Liu et al., 2022).

Based on the wave of globalization and economic development strategy, China should fully control the development of the digital economy from both domestic and international aspects. The domestic market should have a taxation scheme for digital services that is in line with its national conditions to control the digital economy promptly and maximize its impact on the real economy. Overseas, as a player and rule-maker in the global digital economy market, propose a global minimum tax that takes into account the interests of the country while working to narrow the global digital divide and promote sustainable development (SDGs). Based on the development history of unilateral digital service tax and global minimum tax, this paper categorizes and analyzes the design logic of unilateral digital service tax and global minimum tax response strategy of the international community, and proposes the reserve of digital service tax and global minimum tax response plan in China from the basic elements of the tax system.

The contribution of this paper contains the following three main points. The first point compares the development of digital unilateral digital service tax and global minimum tax in various countries so that readers can clearly understand the current status of the global digital economy tax strategies. The second point is to categorize and analyze the response schemes and the logic behind the digital service tax and global minimum tax of each country, so as to provide international experience for the digital service tax schemes of countries that still have not introduced digital service tax and to provide a reference for the response strategies of countries to the global minimum tax. The third point, taking China as an example, proposes a taxation scheme for China’s digital governance and a response scheme for the global minimum tax, which is conducive to the high-quality development of China’s digital economy, and also serves as a reference for other countries with similar situations.

The paper is structured as follows: the second part provides the background information and describes the evolution of the digital service tax, the third part summarizes and analyzes the international experience of tax governance in the digital economy, and the fourth part proposes recommendations for China to cope with the development of the global digital economy, and the fifth part concludes.

## Background information

The use of taxation as a major fiscal policy to regulate the development of the digital business has evolved from theoretical exploration to the creation of a unilateral digital services tax to the negotiation and promotion of a global harmonized taxation scheme by international organizations, which can be divided into the following two stages.

### The origin and development of the unilateral digital services tax

The global development of the digital economy has made a considerable contribution to world economic growth, but it has also posed many challenges to existing economic principles and structures. To address the challenges posed by the digital economy, the Organization for Economic Cooperation and Development (OECD) published its first report on “Addressing the Tax Challenges of the Digital Economy” in 2015 and pointed out that resolving the “mismatch between where value is created and where profits are taxed” is the core of resolving the contradiction between the digital economy and the current tax rules. To resolve the conflict, the international community has successively proposed three options. The first option is to impose VAT or withholding tax on digital economy activities, but this option cannot fully cover the new business models of digital enterprises, such as those non-direct sales that occur in the territory of non-resident enterprises, and those economic activities that do not generate capital flows but can generate economic income. The second option is to reform the current taxation rules from “taxation on the location of the enterprise’s physical permanent establishment” to “taxation of source country income in the source country.” This option has been unanimously accepted by the international community, and the OECD is consulting with countries as pillar I of the two-pillar proposal,^1^ but no specific implementation date has been set yet. The third option is to levy a temporary unilateral digital service tax. The EU proposes to allow member states to formulate a set of digital economy tax principles according to their conditions, which is also the advent of digital service tax on the international stage.

Before the EU proposal, unilateral digital services taxes had appeared in Iceland, South Africa, South Korea, Japan, New Zealand, India, and Australia, but not all under the title of “digital services tax.” In March 2018, the EU proposed a 3% tax on the turnover of Internet giants to combat tax avoidance by multinational companies, but it was opposed by Ireland, the Czech Republic, Sweden, Finland, and other low-rate countries. But it was shelved for the time being due to opposition from low-tax countries such as Ireland, the Czech Republic, Sweden, and Finland. However, this did not discourage the idea of establishing a digital tax in EU countries, and in the same month, the EU issued a proposal for unilateral digital services tax legislation, which would have allowed individual EU member states to tax the effective profits generated by Internet businesses occurring within their borders. Subsequently, countries have introduced digital service taxes in succession, depending on the country, with France introducing the world’s first digital service tax bill on 11 July 2019, followed by Austria, Italy, and Turkey. Also a sovereign country, the tax was strongly opposed by the United States, which has the largest number of Internet businesses in the world. But this has not stopped the global prevalence of unilateral digital service taxes, with multinational internet companies’ tax avoidance issues coupled with the impact of COVID-19, and to help their economies recover as soon as possible, countries such as the United Kingdom, Mexico, Chile, Canada, and Italy have implemented their digital service taxes, and Poland, Brazil, and Nigeria are working on digital tax plans to be introduced in due course.

### The origin and development of the global minimum tax

To recover the tax losses in the digital economy, countries have started to impose unilateral digital service taxes, which has led to the coexistence of multiple tax principles in the international community (Cui, 2019). The coexistence of multiple unilateral digital service taxes increases the risk of double taxation of digital enterprises, and the lack of uniform rules for the division of data benefits undermines the fairness of international tax principles, resulting in international disputes that may undermine global economic growth goals. For example, one of the main reasons for the trade friction between the United States and the European Union is the introduction of a unilateral digital service tax. Therefore, in order to protect the interests of domestic digital enterprises, the United States proposed to replace the “digital service tax” with the “global minimum tax.”

The global minimum tax is an “internationally harmonized” version of the digital services tax. As an alternative to the “unfair unilateral digital tax” of each country, the United States proposed to the OECD to promote the global minimum tax with a global minimum tax rate of 21%. However, considering the interests of low-tax countries such as Ireland, the OECD proposed a tax rate of 12.5%, which is the same as Ireland’s domestic tax rate. In the end, the United States and OECD each took a step back and jointly introduced the current 15% global minimum tax rate tentatively set in Pillar II of the current Statement on a Two-Pillar Solution to the Tax Challenges of a Digitized Economy. On 14 December 2021, the Legislative Template for Addressing the Tax Challenges of the Digitalization of the Economy – Pillar II Global Anti-Base Erosion Rules, jointly developed and adopted by the jurisdictional representatives of all member countries of the BEPS Inclusive Framework, agreed to introduce a 15% global minimum tax domestically in a joint effort to regulate the development of the global digital transformation. Thus, the global minimum tax is a reflection of the current sovereign state-centric global governance model.

As the second-largest economy in terms of the digital economy, China has joined the BEPS inclusive framework, and the introduction of the global minimum tax concerns the interests of our digital enterprises. In the face of the upcoming implementation of the global minimum tax, China should establish the principle of protecting the interests of its digital enterprises as the priority with taking the responsibility of the major participating countries in global governance and use this principle as a guide to learn from the experience of similar countries in collecting and responding to the digital service tax, so as to complete the smooth transformation of China in the digital economy era.

## Analysis and results

It is not difficult to find out that the design of a national digital service proposal or the choice of global minimum tax is not an arbitrary decision, but a reasonable form of national digital service taxation and global minimum tax response under the general direction of international tax reform, following the logic of international taxation principles and making timely and continuous dynamic adjustment according to the national economic situation.

### International experience in the design of unilateral digital service tax

The essence of digital service tax is to use taxation instruments to regulate enterprises where data is a factor of production, in order to satisfy the principle of tax neutrality based on better utilization of its role in driving the economic growth of the country (Rigó and Tóth, 2020). We can reflect on the following three existing digital service tax proposals in the international arena by analyzing the logical mechanism of unilateral digital tax settings in each country.

#### The first type of unilateral digital service tax setting logic

A special digital tax is often imposed, with two starting points, one global and one domestic, and a digital services tax ranging from 2% to 7.5% on income from online advertising, digital interfaces, and sales of user data, which are the main forms of presentation in the domestic digital industry. The setting logic is mostly adopted by developed European economies such as the United Kingdom, France, Italy, Austria, and the Czech Republic, which have low domestic industry chain integrity, no dominant digital technology level, insufficient capacity to provide digital infrastructure, and the scale of the digital economy is mostly located at the middle level in the world, so they mostly choose to lay out the digital economy in the downstream of the value chain, that is, mainly as consumers of digital products and services. They do not have a strong will to support the development of the digital economy, and more often hope to follow the EU digital tax proposal to recover their tax losses while combating the development of the digital economy in other countries, and enhance their position of digital leadership internationally. In addition, this paper finds that although they are all levied under the EU digital tax framework, there is an unspoken logic in the setting of tax rates and domestic starting points in these countries, that is, the tax rates and domestic starting points of digital taxes are decided according to the scale of their digital economy and the degree of economic development, the larger the scale of the digital economy, the lower the digital tax rate, and the more developed their economies, the higher the domestic starting points, as shown in Table 1.

**Table 1:** First option unilateral digital taxation scheme for major economies.

	Digital ranking	GDP/person	Tax type	Tax base and taxation scope	Tax rate	Taxation threshold
EU	–	22.8	DST	Targeted advertising, digital interface, and sales of user data revenues	3%	Global revenue of 750 million, 40 Euros EU-wide
France	Top 16	27.1	DST	Digital interface, targeted advertising, and transmission subscriber data service revenues	3%	Global revenue of 750 million, 25 million Euros domestically
Italy	Top 16	22	DST	Targeted advertising, digital interface, and subscriber data transmission revenues	3%	Global revenue of 750 million, 5.5 million Euros domestically
Spain	Top 16	20.1	DST	targeted advertising, multi-terminal digital interfaces, and transmission of user data revenues	3%	Global revenue of 750 million, 3 million euros domestically
Austria	16–32	33.7	DST	Online advertising business in B2B mode revenues	5%	Global revenue of 750 million, 25 million Euros domestically
Czech	16–32	15.9	DST	Targeted advertising, subscriber data transmission, and multi-terminal digital interface revenues	5%	Global revenue of 750 million, 100 million kroner domestically, and more than 200,000 users
Poland	16–32	10.8	DST	Online advertising revenues	5%	750 million Euros worldwide, 5 million Euros domestically
Turkey	16–32	6	DST	Targeted advertising, social media, and digital interface services revenues	7.5%	750 million Euros worldwide, 20 million Turkish Lira domestically

World ranking of the digital economy is from “White Paper on Global Digital Economy – A New Dawn of Recovery under the Impact of the Epidemic” released by the China Academy of Information and Communications Technology in 2020, with the top 16 being the top and 17–32 being medium; GDP per capita data is from the IMF release in 2020 and is measured in RMB 10,000. The dollar to the yuan exchange rate is set at 7.

#### The second type of unilateral digital service tax setting logic

Modifying the original tax to levy a digital services tax to include the main forms of presentation of the domestic digital industry in the scope of the original tax, which is usually levied on foreign businesses, with only a domestic annual income threshold. The representative countries of this scheme are Asian countries such as Japan and South Korea, where the domestic industry chain is more complete, the scale of digital technology and digital economy is at the forefront of the world, and the country vigorously supports digital technology research and development and infrastructure construction, so the introduction of digital service tax is very cautious and prefers to explore digital service tax in the form of existing taxes, and use tax policies to better assist traditional industries in the transition period of their transformation to digitalization. In the transition period of traditional industries, tax policies can be used to better support the efficiency of the industry chain and achieve economic growth goals (Sun et al., 2016; Li et al., 2022). As shown in Table 2, the coverage of digital service tax in these countries is relatively narrow at this stage, and the minimum tax rate of existing taxes is usually extended, e.g., Japan’s digital tax rate is set at 8% when the consumption tax rate on imports is 8% (generally applicable to food and beverage products), and Korea’s digital tax rate is set at 10% when the VAT rate is 10%.

**Table 2:** Digital taxation scheme for major economies in the second scenario.

	Tax type	Tax base and scope of taxation	Tax rate	Taxation threshold
Japan	Consumption tax	Digital business owners import digital sales	8%	Annual 10 million yen
Korea	VAT	Value-added B2C digital services of foreign companies, such as online advertising and cloud computing	10%	Korea VAT threshold.
Thailand	VAT	Value-added foreign e-commerce, online advertising, and digital platforms	7%	over 1.8 million baht domestically
Indonesia	VAT	Value-added from streaming services, apps, and digital games	10%	Rp 600 million domestically or 12,000 annual users

#### The third type of unilateral digital service tax setting logic

The initial levy is in the form of a pre-existing tax, which is then reformed to a “new tax” logic. India is a typical representative country of such a scheme. In recent years, India’s digital market has been expanding, coupled with its years of OEM for the United States, its digital technology has been enhanced, and the scale of the digital economy has been growing rapidly. It wants to recover the taxes of being a “market country” and move to a “resident country.” Therefore, its first “equalization tax” is based on the practice of Asian countries to explore the taxation of online advertising at a rate of only 6%. Later, when India encountered the impact of COVID-19 and was in dire need of tax revenue to help its economy recover, it chose to follow the practice of most international countries, that is, to improve its digital tax under the logical framework of European countries’ digital service tax settings, expanding the scope of taxation to include e-commerce and sales of user data, etc. In addition, the tax rate has been reduced and the threshold has been raised, as shown in Table 3.

**Table 3:** Third scenario digital tax scenario for major economies.

	Digital ranking	GDP/person	Tax type	Tax base and taxation scope	Tax rate	Taxation threshold
The first equalization tax of India	–	–	Sales tax	Foreign online advertising	6%	Extremely low
The second equalization tax of India	Medium	1.3	Sales and service tax	Online advertising, e-commerce, and sales of user data revenues provided by foreign companies	2%	20 million rupees domestically

### The logic enlightenment of China’s unilateral digital services tax

**Figure 1:**
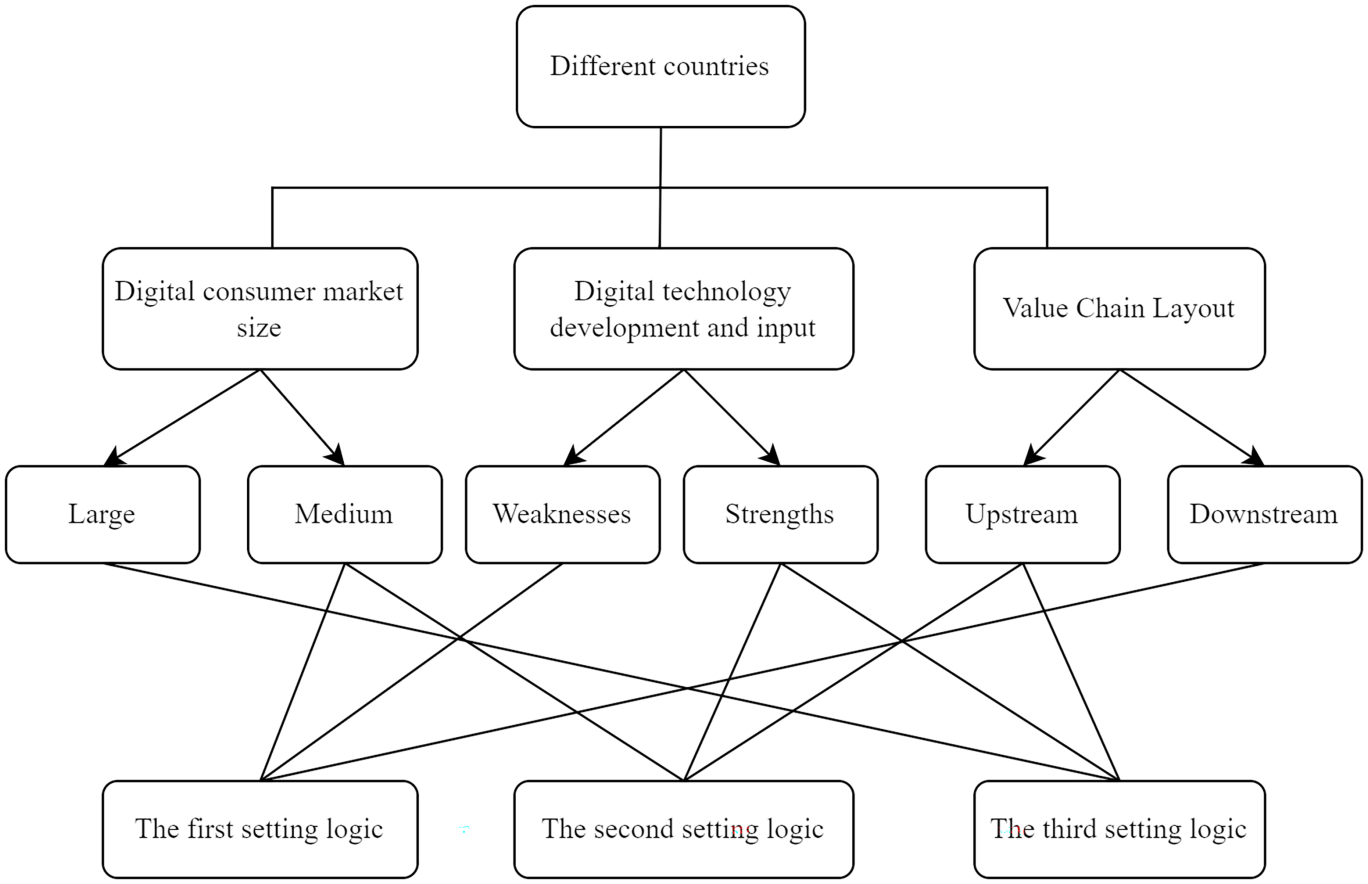
Three logics of unilateral digital service tax design.

Summarizing the above three options, we can conclude Figure 1 from three dimensions: digital consumption market size, digital technology R&D and investment capacity, and value chain layout. Countries that choose the first option to levy digital services tax prefer to lay out the digital economy in the downstream of the value chain with their economic characteristics, and countries that choose the second option prefer to lay out the digital economy in the upstream of the industry chain, while India in the third option prefers to upgrade the digital economy from the downstream of the value chain to the upstream of the value chain.

China is still among the developing countries, with a relatively complete domestic industrial chain and a real economy that is the lifeblood of the national economy, and for many years China has been committed to the road of high-quality development of the real economy. The rapid development of the digital economy provides an opportunity for China’s industrial chain to improve efficiency and the prosperity of the real economy. As the country with the second-largest number of the world’s top 100 enterprises and the leading digital economy in terms of scale and development speed, China has been committed to shifting from digital consumption to digital technology research and development and digital infrastructure construction and has absolute international competitive advantages in supporting domestic economic growth through the digital economy, but because of the “big trees attract wind,” it is easy to fall into However, it is prone to international trade disputes due to its large size, so it should be more cautious in the introduction of digital service tax. In this paper, based on the strategic goal of digital economy development and international trade pattern, China’s digital service tax can learn from India’s experience and refer to the third option, which is to first tax only a small percentage of the digital industry in the form of original tax to explore its digital service tax policy and then follow the international digital tax setting rules to expand the tax scope and adjust it dynamically according to the evolution of the digital economy.

### International experience of global minimum tax response

As an internationally harmonized version of the digital services tax, the Global Anti-Base Erosion (GloBE) rules aim to address the legacy of base erosion and profit loss that has been exacerbated in the digital economy. According to the Pillar II Legislative Template, the GloBE rules require multinational corporations with annual consolidated group revenues of EUR 750 million to pay at least 15% of their effective corporate income tax and to comply with the Income Inclusion Rule and the Undertaxed Payment Rule. Since it is under the BEPS Pillar II proposal framework, it is also subject to the Switch-over Rule^2^ and the Subject To Tax Rule.^3^ Compared with the unilateral digital service tax, the global minimum tax has more obvious political attributes and is an important part of the international tax reform, which is related to the goal of stable growth of the world economy and the economic interests of each country, and different countries have different attitudes and response strategies toward its introduction.

#### The first response strategy

Agree to introduce a global minimum tax domestically to benefit domestic multinational enterprises from the tax rate. The United States is the representative. The United States is an opponent of global governance, but to solve the dilemma of “unreasonable” taxation faced by its multinational companies, it has introduced a global uniform tax system instead of unilateral measures of each country, and at the same time proposed that the development of the global digital economy and tax avoidance by multinational companies should be regulated at a higher rate (21%). This is mainly for two reasons. On the one hand, the Biden administration intends to raise the United States domestic corporate tax rate from 21% to 28% due to the new infrastructure plan, which will, to a certain extent, weaken the international competitiveness of the United States tax rate and cause United States domestic companies to shift their profits, offsetting the positive impact of the new infrastructure plan on the long-term economic development of the United States. On the other hand, the global minimum tax rate of 21% is exactly the same as the current United States domestic tax rate, which can play a good role in maintaining or even improving the competitiveness of international tax rates and thus reducing tax avoidance losses, regardless of whether the new infrastructure plan is passed by Congress.

#### The second response strategy

Agree to introduce a global minimum tax domestically, and fight for the benefits of domestic multinationals in terms of other levy rules. The vast majority of BEPS Inclusive Framework members are represented by global governance leaders.^4^ Among these countries, those with higher domestic corporate income tax rates, such as the United Kingdom, often propose to retain the “safe harbor” principle in their original tax systems, as proposed in the Pillar II proposal, which has now been adopted in the Pillar II Legislative Template as an exclusion mechanism from the GloBE rules. Unlike these countries, countries with lower corporate income taxes look more to the OECD to take the initiative and propose a more reasonable response for their interests. Ireland, for example, has a domestic tax rate of 12.5%, and the introduction of a global minimum tax would deprive it of its main means of attracting foreign investment, and its proposal to join the global minimum tax is more due to pressure from international organizations. Since the global minimum tax would have a greater uncertain impact on their domestic economies, the more consideration they would need to give to the details of the global minimum tax, the more they would look forward to a global minimum tax that “takes into account their circumstances” when they join the two-pillar proposal.

#### The third response strategy

**Figure 2:**
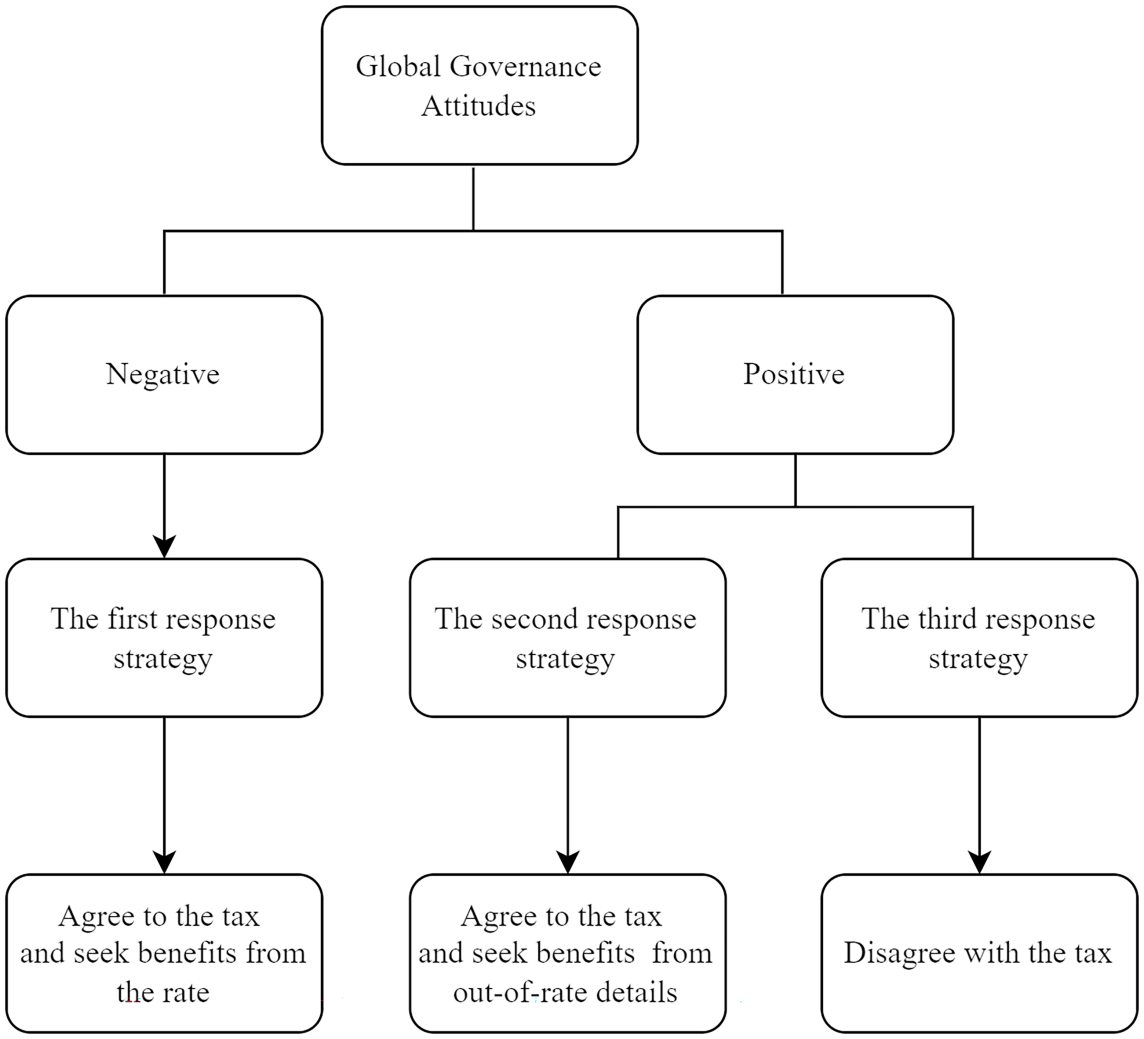
Three response strategies of the global minimum tax.

Do not agree to introduce a global minimum tax at home. Most of the countries/regions in this part are very small economies, which play more of a follower and beneficiary role in global governance shown in Figure 2. A low tax rate is their main means to attract foreign investment, and the introduction of a 15% global minimum tax will affect their low-tax advantage and squeeze domestic tax revenue. That is why they have not joined the global minimum tax agreement so far.

### The response strategy enlightenment of China’s global minimum tax

To address the tax challenges of the digital transformation, China should not only consider its tax management strategy for the digital economy but also actively respond to the global tax reform for the digital economy. Adhering to the attitude of actively participating in global governance, China has joined the global minimum tax proposal and agreed to implement the global minimum tax domestically by 2023. Currently, our domestic corporate income tax rate is 25%, which is lower than the global minimum tax rate of 15%. Therefore, according to our global minimum tax response strategies derived above, China should hold the second type of attitude, learn from the practice of countries with high tax rates, measure and list the impact of the global minimum tax on our country, formulate corresponding solutions, and propose mechanisms to retain or exclude the global minimum tax rules that fit both our economic development strategy and our position as one of the main advocators in the global governance system.

## Discussion

From the summary above, we can see that tax governance policy for the digital economy is not only an economic instrument but also requires multiple considerations at the political level. Whether it is a unilateral digital service tax or a global minimum tax, countries that subsequently introduce and join it tend to formulate it within the framework of international design and response logic, taking into account their economic characteristics. The existing provisions of the unilateral digital services tax are instructive for China’s tax management of the digital economy, while the international response to the global minimum tax is a valuable reference for China’s strategies to safeguard its interests in the context of international tax reform while strengthening its position in the global governance system.

### China’s tax governance strategies for the digital economy

#### Initial reliance on VAT for taxing digital products and services

Unlike traditional enterprises, digital enterprises are “virtual enterprises” that rely on digital technology to map tangible enterprises into the network and can partially or globally simulate the behavior of tangible enterprises, and they have certain vulnerabilities as new types of enterprises. Based on China’s strategic goal of giving priority to supporting the development of the digital economy, taxing digital enterprises at the initial stage of their growth will, to a certain extent, increase their operating costs and discourage their owners from establishing and developing digital enterprises, especially in the absence of appropriate tax rules, which is a threat to the long-term development of China’s digital economy. Secondly, the high time cost of the initial introduction of the digital services tax not only requires consideration of its friction with existing taxes from the perspective of different digital products but also places higher demands on the digital skills of tax practitioners and tax authorities.

However, tax collection is beneficial to further consolidate and build digital infrastructure and further establish the foundation for the development of the digital economy. Therefore, China should actively explore but cautiously try out a taxation scheme for the digital economy. The first introduction of a digital service tax can refer to the third design scheme, which first draws on the experience of Asian countries and attaches to existing taxes to tax digital enterprises. For the choice of existing taxes, VAT can be considered as an alternative tax following the logic set by Asian countries. VAT is currently the largest tax in China, and there is already a relatively mature VAT collection and management system in China. For digital products and services with a higher degree of concealment, it is more in line with the principle of efficiency to tax the digital economy in the form of VAT. From the perspective of VAT’s characteristics, VAT has a broader coverage and is a behavioral tax, which has the feature of non-duplication of taxation, which ensures the principle of tax fairness to a certain extent to mitigate the negative impact of digital tax. In terms of implementation, a digital VAT can be implemented on a pilot basis to observe the impact of tax shocks on digital enterprises in the pilot areas, so as to limit the adjustment to a manageable extent and pave the way for nationwide digital tax reform.

#### Gradually expand the scope of the tax from a small percentage of the digital industry to include the major digital industries

The volume of China’s digital economy industry is expanding day by day, and the tax levy is favorable to China’s industrial digital economy penetration rate of 21% and service industry penetration rate of 40.7% in 2020, according to the China Academy of Information and Communications Technology (2020). If a tax is imposed on digital products and services on a large scale, it will accompany a considerable amount of tax revenue while also throwing the control of the digital tax policy effect out of the hands, and subsequently failing to achieve hedging or guiding the industry chain to improve efficiency by adjusting monetary and fiscal policies, which risks endangering the economy. Therefore, it is important to set the tax scope of the first digital tax.

Drawing on the third design option, a certain industry with obvious digital characteristics but not a high proportion of the economy is first used as a pioneer industry, such as the online advertising industry. Internet advertising is a typical representative of the digital economy industry, and its market scale in China is rising year by year, accounting for about 0.9% of China’s GDP. The trial implementation of digital service tax with the advertising industry can explore the perfect tax legislation rules while not having a large adverse impact on the development of China’s digital economy. After reforming the digital service tax, the main presentation form of China’s digital economy into digital platforms and Internet search engines will also be included in the scope of taxation, striving to maximize the fairness of the business environment. The platform economy is the main presentation form of the digital economy, which completes all kinds of economic activities through data-driven, platform-supported, and network collaboration, and is the general name of various economic relationships on digital platforms, both which and Internet search engines account for a considerable proportion of China’s digital economy industry, and both of which have opened up new paths of economic growth by improving the matching of supply and demand (Milovanov, 2022). The inclusion of them into the scope of digital service tax for reasonable regulation is in line with their own “digital” characteristics and their digital scale in China, reducing the vicious competition caused by their disorderly development and the squeeze on traditional industries due to the lack of fairness, and is an important channel to protect consumer information security in the digital era.

#### Rely on the existing VAT system to set the tax rate and starting point, and follow the international law to reform after expanding the scope of taxation

The tax rate and the starting point are important components of the tax, which not only determine the depth of impact on digital enterprises but also concern the goal of optimizing the tax business environment. According to international experience, China can first tax the subject digital products and services under the existing VAT system. The online advertising industry is currently included in the scope of VAT in China and the applicable tax rate is 6%. Therefore, China can consider raising the applicable tax rate to the basic rate of 13% and refer to the “Rules for the Implementation of the Provisional Regulations of the People’s Republic of China on Value-Added Tax” to set the starting threshold.

After the timely reform to expand the scope of taxation, the new digital tax rate and the starting point will be set according to the level of development of the digital economy and the international ranking of the degree of economic development. According to international experience, the larger the scale of the digital economy in the country, the lower the tax rate can be. For China, the reformed tax on digital products and services can be lower than the lowest level of the existing unilateral digital service tax rate of 2%. On the one hand, to protect the development of the country’s digital economy, the initial trial digital tax minimizes the tax rate to suppress the negative impact of the digital tax. On the other hand, the United States, as the first country in the digital economy, has not introduced a digital service tax, and China, as the second-largest country in the digital economy, should have the right to set the lowest tax rate. On the choice of the starting point, international law requires that the more developed the country’s economy is (the higher the GDP *per capita*), the higher the starting point. Therefore, China can set the starting point between the digital tax thresholds of countries with similar economic development levels as ours. According to IMF statistics, China’s (mainland China) GDP *per capita* in 2020 is 82,600 yuan, which is located between Poland and Turkey (Table 1), so our reformed digital tax threshold can be located between the thresholds of these two countries, that is, in the range of 20–35.5 million yuan. No matter whether it is the tax rate or the starting point, the final plan needs more consideration in light of the digital economy and national economic development stage and specific characteristics of China at that time.

### China’s global minimum tax response program

#### Active participation in global minimum tax development

As one of the digital leaders, China should sort out the contradictions between the existing tax system and the global minimum tax, actively formulate solutions, and propose to the OECD Chinese proposals on the details of the global minimum tax from the perspective of the exclusion mechanism or the retention mechanism, to protect its interests first and foremost to reflect the role of a major country.

On the one hand, the government should actively organize the calculation of the domestic impact of the global minimum tax, sort out the list of conflicts between the global minimum tax and the existing economic policies of the country, and think of solutions for each “conflict.” Some of the “conflicts” can be offset by actively adjusting domestic policies, for example, for those enterprises that enjoy China’s tax support with an effective tax rate lower than 15%, a set of VAT tax incentives can be set up to hedge their impact. For those “conflicts” that cannot be offset, they can be proposed to the OECD as exclusion clauses or reservation mechanisms, such as the expiring bilateral tax agreements in the “One Belt, One Road,” the exemption system for equity participation in Hainan Free Trade Port and various tax incentives.

On the other hand, as an advocate of the community of human destiny and the country with the largest contribution to the world’s major economic growth rates, China’s global minimum tax proposal also needs to take into account the world economic landscape. The global minimum tax rate is temporarily set at 15%, which is much lower than our corporate income tax rate of 25%, but close to our tax rate in Hong Kong and higher than the tax rates of many small economies in the world. In other words, countries are allowed to set their own global minimum tax rates within a certain tax range to ease the sudden pressure of tax increases on their domestic foreign investment.

#### Actively improve the domestic layout of the global minimum tax levy

The taxation department has formed a global minimum tax task force, made a good division of labor within the group, formulated a global minimum tax collection and management path, and deployed tax collection and management arrangements in advance to ensure the efficient implementation of China’s global minimum tax.

First, follow up on the progress of the global minimum tax in real-time, analyze the tax collection and administration arrangements of the countries that have already introduced it, learn from international experience, and study a reasonable tax collection and administration system.

Secondly, to improve the digital technology level of the tax department, accelerate the digitalization process of the tax department, and prepare for the layout and implementation of the global minimum tax collection system. As a resident country, the digitalization of the taxation department is a future development trend, and the convenience of the digital system can largely compress the time and reduce the cost of tax collection and administration, which is a feasible way to improve the efficiency of our taxation department. As a source country, due to the “uniqueness” of production factors and the “hidden” nature of economic activities of digital enterprises, China needs to do some upgrading and transformation of the original offshore taxation system. These include improving the data transmission, sharing, and verification system, updating the list of offshore enterprises subject to taxation in China, adding an effective tax rate and tax burden estimation system based on GloBE rules, and improving the security of the tax payment system and monitoring system.

Furthermore, a global minimum tax task force will be set up to educate Chinese digital enterprises, advise them to make reasonable tax planning and economic activity arrangements as early as possible, understand the impact of the global minimum tax on different types of digital enterprises, and the difficulties they encounter through regular seminars, and listen to their suggestions on how to cope with the global minimum tax so that the government and enterprises can work together to safely survive this international tax reform in the digital economy. We also seize this opportunity to promote the international competitive advantage of China’s digital enterprises to a new level.

## Conclusion

The digital transformation has brought opportunities for economic growth and at the same time posed direct challenges to traditional tax principles, with traditional and digital businesses facing a lack of fairness and an inefficient business environment. Taxes are expected to be an important economic tool in addressing these challenges. Countries have introduced temporary digital service tax plans and the international community has negotiated global minimum tax development strategies, all of which aim to solve the problem of incompatibility between traditional tax principles and the digital economy. The global minimum tax provisions scheduled to be implemented by the end of 2023 have rendered bilateral tax agreements and tax incentives in many countries ineffective. Therefore, before the international taxation scheme for the digital transformation is finalized, countries should actively participate in the formulation of international taxation rules to reduce the impact of unilateral digital service taxes on multinational enterprises in other countries, and at the same time actively reserve their tax management strategies for the digital business models, to make full use of the digital economy as a powerful tool to improve the efficiency of industrial chains and vigorously promote economic prosperity during the digital transformation period.

## Data availability statement

The datasets presented in this study can be found in online repositories. The names of the repository/repositories and accession number(s) can be found in the article/supplementary material.

## Author contributions

All authors listed have made a substantial, direct, and intellectual contribution to the work and approved it for publication.

## Funding

Thanks to the support of the key project of the National Social Science Foundation of China (21AGL012).

## Conflict of interest

The authors declare that the research was conducted in the absence of any commercial or financial relationships that could be construed as a potential conflict of interest.

## Publisher’s note

All claims expressed in this article are solely those of the authors and do not necessarily represent those of their affiliated organizations, or those of the publisher, the editors and the reviewers. Any product that may be evaluated in this article, or claim that may be made by its manufacturer, is not guaranteed or endorsed by the publisher.

## Footnotes

^1^The two-pillar solution is an international solution proposed by the OECD to address the tax challenges posed by the digital economy, which requires multi-country agreement and then global implementation. Pillar I aims to address the issue of profit sharing by multinational corporations in the digital economy. Pillar II aims to reach a uniform tax administration scheme for the global digital economy to alleviate the tax avoidance problem of multinational corporations, i.e., to impose a uniform corporate income tax at the lowest rate on global multinational corporations, but the tax rate and other tax details have been delayed.

^2^The SOR is a subsidiary rule of the IIR that applies when the local effective tax rate on profits or gains derived from a foreign permanent establishment or foreign real estate (real estate owned by a non-resident) is lower than the minimum tax rate and allows the resident country to convert the exemption in the tax treaty to a credit.

^3^The STTR rules are a supplemental application of the UTPR. It provides that the source country is entitled to withholding tax at the minimum STTR rate on cross-border payments of interest, royalties and other specified payments.

^4^From the database of the World Bank’s Worldwide Governance Indicators.
